# Pathogenic mutations in UBQLN2 exhibit diverse aggregation propensity and neurotoxicity

**DOI:** 10.1038/s41598-024-55582-9

**Published:** 2024-03-13

**Authors:** Nathaniel Safren, Thuy P. Dao, Harihar Milaganur Mohan, Camellia Huang, Bryce Trotter, Carlos A. Castañeda, Henry Paulson, Sami Barmada, Lisa M. Sharkey

**Affiliations:** 1 Departments of Biology and Chemistry, Syracuse University, Syracuse, NY 13244 USA; 2 Department of Neurology, University of Michigan, Ann Arbor, MI 48109-2200 USA; 3 Cellular and Molecular Biology Program, University of Michigan Medical School, Ann Arbor, MI 48109 USA; 4 Michigan Neuroscience Institute, University of Michigan, Ann Arbor, MI 48109-2200 USA; 5 Present Address: Department of Neurology, Northwestern University Feinberg School of Medicine, Chicago, IL 60611 USA

**Keywords:** Molecular neuroscience, Neurodegeneration

## Abstract

The ubiquitin-adaptor protein UBQLN2 promotes degradation of several aggregate-prone proteins implicated in neurodegenerative diseases. Missense *UBQLN2* mutations also cause X-linked amyotrophic lateral sclerosis (ALS) and frontotemporal dementia (FTD). Previously we demonstrated that the liquid-like properties of UBQLN2 molecular assemblies are altered by a specific pathogenic mutation, P506T, and that the propensity of UBQLN2 to aggregate correlated with neurotoxicity. Here, we systematically assess the effects of multiple, spatially distinct ALS/FTD-linked missense mutations on UBQLN2 aggregation propensity, neurotoxicity, phase separation, and autophagic flux. In contrast to what we observed for the P506T mutation, no other tested pathogenic mutant exhibited a clear correlation between aggregation propensity and neurotoxicity. These results emphasize the unique nature of pathogenic *UBQLN2* mutations and argue against a generalizable link between aggregation propensity and neurodegeneration in UBQLN2-linked ALS/FTD.

## Introduction

A member of the UBA-UBL family of ubiquitin adaptor proteins, UBQLN2 (Ubiquilin 2) acts as a substrate recognition component for the proteasome and the autophagy-lysosome system, enabling it to target misfolded or damaged proteins for degradation^1–4^. Abnormalities in UBQLN2 expression and function are linked to numerous neurodegenerative diseases, including Alzheimer’s, Parkinson’s, amyotrophic lateral sclerosis (ALS) and frontotemporal dementia (FTD)^5–10^. *UBQLN2* mutations can also directly cause neurodegeneration, most notably X-linked ALS/FTD ^5,6,11–14^.

UBQLN2 reversibly undergoes liquid–liquid phase separation (LLPS), forming molecular assemblies that may participate in cellular stress responses^15–18^. Biomolecular condensates, hypothesized to form via phase separation mechanisms, are dynamic cellular compartments that allow for the enrichment of proteins and other molecules dedicated to specific cellular functions^19,20^. Cellular condensate formation is associated with many biological processes including transcription, translation, signal transduction, and protein folding. Recently, interest in condensates has increased because of their potential role in the pathogenesis of ALS, FTD, Huntington’s disease and other neurodegenerative conditions^21–24^. In particular, disruption of normal condensate formation or maintenance may provide a mechanistic link to the development of these diseases ^20,21^.

Mirroring the capacity for RNA binding to tune the phase separation of RBPs, UBQLN2 LLPS is affected by ubiquitin binding in vitro^15^. This behavior raises the intriguing possibility that UBQLN2 phase separation plays a critical role in its function as a ubiquitin-dependent proteasomal shuttle protein. However, these studies used supraphysiological concentrations of recombinant UBQLN2 and ubiquitin in an environment free of other cellular components.

Previously we demonstrated that the material properties of UBQLN2 liquid-like molecular assemblies within cells are altered by the pathogenic P506T mutation^18^. This missense mutation impairs UBQLN2 mobility within condensates while also increasing UBQLN2 aggregation and toxicity upon overexpression in rodent neurons. Pathogenic *UBQLN2* mutations, including P506T, cluster within a proline rich PXX domain, although several pathogenic variants exist outside of this region^14,25,26^. Because the different domains of UBQLN2 impart distinct ability to drive or modulate UBQLN2 phase separation in vitro^15^, we sought here to characterize the phase separation propensities and aggregation propensities of UBQLN2 variants harboring mutations falling both within and outside the PXX domain in cells. Given previous evidence of macroautophagy deficits and neuronal loss in UBQLN2-related disease models^27–29^ we also evaluated autophagy flux and neurotoxicity in primary neurons. These studies uncovered unique effects of each mutation on condensate dynamics, *in cellulo* puncta formation and neurotoxicity, arguing against a single, conserved pathogenic mechanism associated with UBQLN2 mutations.

## Results

### UBQLN2 mutations alter granule fluidity

**Figure 1:**
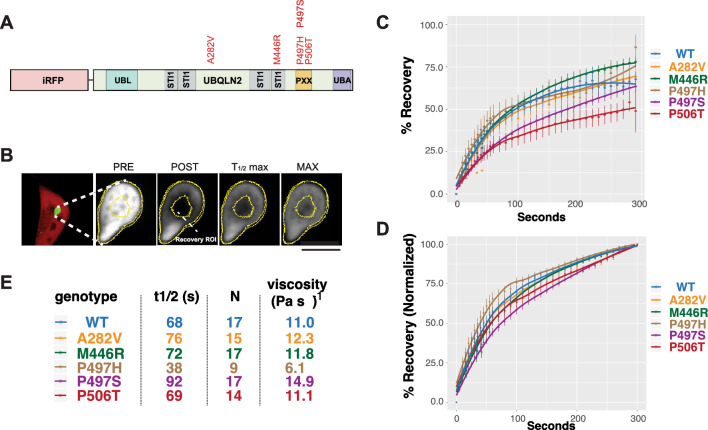
UBQLN2 mutations differentially affect granule fluidity. (**A**) Schematic showing the GFP- or iRFP-tagged UBQLN2 constructs used in the current study. The tested human ALS mutations are highlighted in red. (**B**) Fluorescence recovery after photobleaching (FRAP) of GFP-UBQLN2 granules. GFP-UBQLN2 and mApple were co-transfected in HEK293 cells and imaged the next day. The region of interest (ROI) specifies the photobleached area and the area analyzed for recovery. Scale bars = 5 µm (**C**) Time-dependent fluorescence recovery for UBQLN2 variants. Each point represents the mean recovery of at least 9 granules, with error bars representing SEM. A one-way ANOVA found significant effect of genotype on mobile fraction F = 3.234, *p* = 0.006, and t1/2 max F = 5.526, *p* = 5.44E−5. Tukeys post-hoc test found a significant difference in the mobile fraction of WT and P506T granules. (**D**) The recovery curves depicted in *C* but with the maximum recovery (mobile fraction) normalized to 1, highlighting recovery kinetics. (**E**) Table summarizing the half-maximal recovery (t_1/2_), granule viscosity, and number of granules (N) assessed for each genotype.

We first determined the effect of five pathogenic UBQLN2 mutations on granule fluidity. UBQLN2-A282V, UBQLN2-M446R, UBQLN2-P497S, UBQLN2-P497H, UBQLN2-P506T were chosen to include mutations both within the PXX domain, where ALS/FTD mutations are most commonly located, as well as outside of the PXX domain. WT HEK293 cells were co-transfected with GFP-tagged UBQLN2 constructs (Fig. 1A) and mApple to visualize cells. Individual UBQLN2 granules were identified by confocal fluorescence microscopy. We measured granule size in cells expressing each of the variants and found that there was no relationship between granule size and genotype (Figure S1). Similar size granules were chosen across UBQLN2 constructs and photobleached using high-intensity illumination (Fig. 1B–D). Fluorescence recovery after photobleaching (FRAP) was measured at regular intervals, allowing us to calculate the time to half-maximum recovery (t_1/2_ max) and viscosity for each granule (Fig. 1E). Our analysis found that the rate of recovery for each individual mutant was not significantly different from WT. As previously demonstrated^18^, the UBQLN2-P506T mobile fraction was significantly lower than that of WT UBQLN2. All other UBQLN2 mutants were similar to UBQLN2-WT.

**Figure 2:**
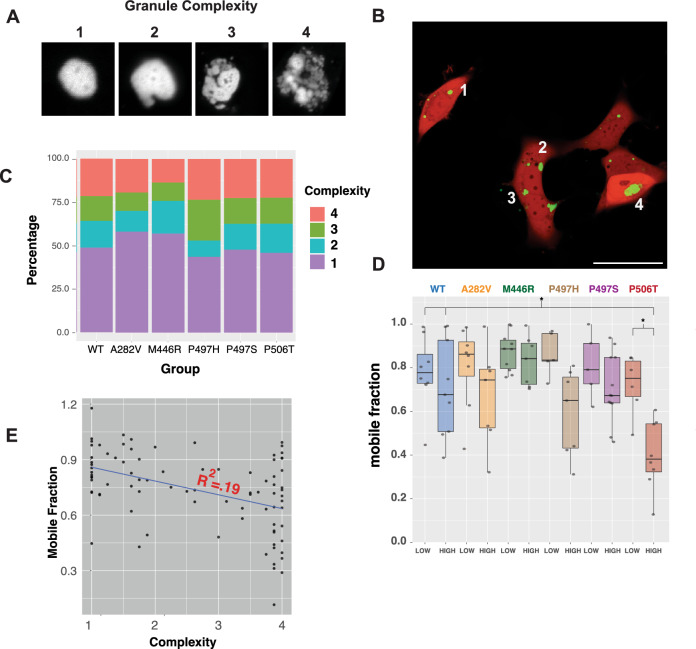
Granule complexity and mobile fraction are correlated in UBQLN2-P506T granules. (**A**) Representative granules depicting each of four levels of increasing complexity used in panels *B-D*. (**B**) Example of scoring complexity across genotypes. HEK293 cells were co-transfected with mApple and GFP-UBQLN2 and imaged the next day. Fluorescent images are of cells expressing GFP-UBQLN2 (green). White numbers indicate examples of granules in each complexity category. Each granule in at least 6 images per genotype was scored for complexity after blinding the genotype of each image. (**C**) Cumulative frequency of granules of each complexity across genotype. The number of granules, mean and median complexity, and standard deviation is reported in Table 1. (**D**) Relationship between genotype, complexity and recovery. Granules with a mean complexity score of 1 and 2 were grouped into “low complexity” and those with a score of 3 or 4 as “high complexity”. Each dot represents an individual granule. * denotes *p* < .05, one-way ANOVA with Sidak’s multiple comparisons test. Table 2 summarizes this analysis. (**E**) Relationship between granule complexity and mobile fraction irrespective of genotype.

We also analyzed the relationship between granule complexity and UBQNL2 mobility. Although GFP-UBQLN2 puncta vary in appearance, we previously observed that mutant UBQLN2-P506T on average forms more irregularly shaped puncta than UBQLN2-WT, and this increased complexity correlated inversely with the amount of fluorescence recovery^18^. Here, we scored granule complexity across genotypes (Fig. 2A, B) and assessed the distribution of granules falling into each complexity category (Fig. 2C). Overall, no UBQLN2 mutation significantly affected the complexity of granules in comparison to UBQLN2- WT. However, the mean complexity of UBQLN2-M446R and UBQLN2-A282V granules trended low, while UBQLN2-P497H displayed a trend towards higher complexity compared to UBQLN2-WT.

We also investigated the relationship between genotype, complexity, and internal mobility of UBQLN2 granules. UBQLN2-P506T showed a positive correlation between increased granule complexity and reduced FRAP recovery as previously observed^18^. No other mutants displayed such a correlation, however (Fig. 2D, E). These results suggest that *UBQLN2* mutations affect granule fluidity in a complex manner that is dependent on both the location and amino acid change of specific UBQLN2 mutations. Additionally, among granules scored as high complexity, P506T granules had reduced recovery vs. WT high-complexity granules. This observation suggests that while granules may appear similar via microscopy, each mutation may be affecting granule structure and mobility in subtle ways that do not affect granule appearance.

Granule appearance via microscopy cannot differentiate between liquid-like condensates and stable aggregates. To determine whether there were differences among the different genotypes in the proportion of expressed UBQLN2 protein existing as soluble condensates versus insoluble aggregates we performed a detergent solubility assay^30^ to compare the aggregation propensity of UBQLN2 variants (Figure S2). The results show that UBQLN2 is expressed as a mixture of soluble protein, condensates, and aggregates. No significant differences were detected among variants.

**Table 1:** Number of granules, mean and median complexity, and standard deviation.

Group	n	Mean	Median	SD
WT	78	2.089744	2	1.229358
A282V	301	1.910299	1	1.206342
M446R	278	1.805755	1	1.090674
P497H	64	2.265625	2	1.250298
P497S	94	2.117021	2	1.23442
P506T	113	2.132743	2	1.221127

**Table 2:** Relationship between UBQLN2 genotype, granule complexity and fluorescence recovery.

Group	n	Mean	Median	SD	Versus within genotype low complexity	Versus WT of same complexity
WT_low	8	0.779	0.777	0.168	N/A	N/A
WT_high	9	0.707	0.677	0.224	Non-significant	N/A
A282V_low	8	0.805	0.862	0.188	N/A	Non-significant
A282V_high	7	0.67	0.744	0.224	Non-significant	Non-significant
M446R_low	10	0.873	0.886	0.088	N/A	Non-significant
M446R_high	7	0.83	0.842	0.115	Non-significant	Non-significant
P497H_low	5	0.863	0.835	0.1	N/A	Non-significant
P497H_high	7	0.593	0.65	0.198	Non-significant	Non-significant
P497S_low	5	0.81	0.791	0.149	N/A	Non-significant
P497S_high	12	0.706	0.673	0.154	Non-significant	Non-significant
P506T_low	6	0.723	0.751	0.136	N/A	Non-significant
P506T_high	8	0.401	0.381	0.159	**Significant**	**Significant**

Granules with a mean complexity score of 1–2 were grouped into “low complexity”, and those with a score of 3–4 as “high complexity”. mean = mean mobile fraction, n = the number of granules observed in each complexity category per genotype. For each genotype, mean mobile fraction was compared between low complexity and high complexity granules. Mean mobile fraction was also compared to WT granules of like-complexity. Significance is reported for those comparisons that reached a *p* value < 0.05 using Sidak’s multiple comparions test.

Significant values are in bold.

### UBQLN2 mutations differentially modulate LLPS

**Figure 3:**
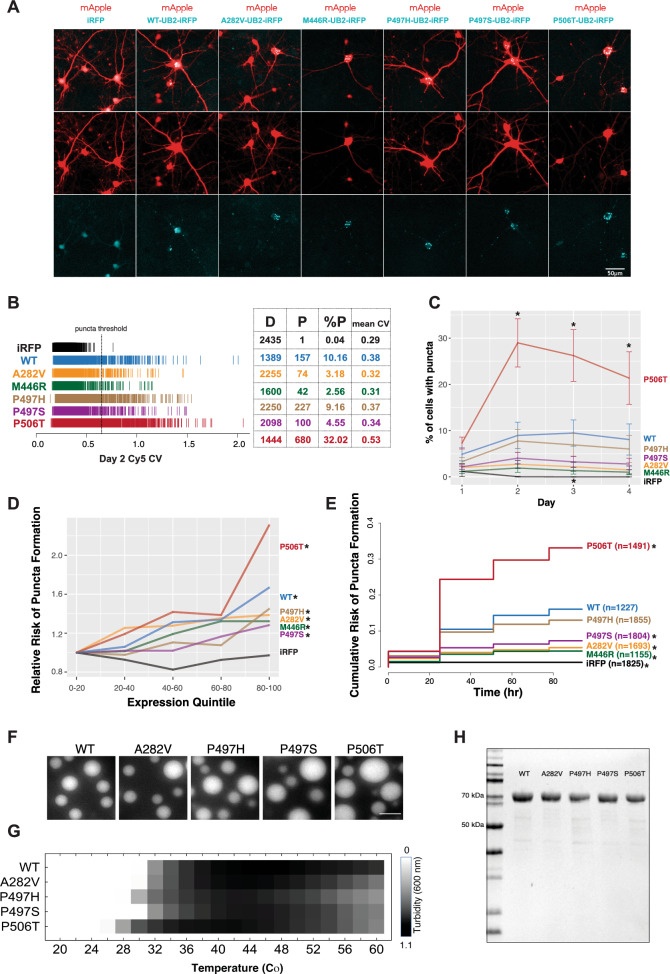
UBQLN2 mutations differentially affect self-assembly. (**A**) Primary rat cortical neurons transfected at DIV4 with near-infrared fluorescent protein (iRFP) or iRFP-tagged UBQLN2 constructs. Neurons were co-transfected with mApple to serve as a morphology marker. Images acquired 2 days after transfection show that iRFP fluorescence remains diffuse while all iRFP-UBQLN2 proteins form cytoplasmic puncta. Scale bar = 50 µm. (**B**) Single cell coefficient of variation (CV) of Cy5 fluorescent intensity in neurons imaged 2 days after transfection. Each hash mark represents an individual neuron. Neurons above the previously identified aggregate threshold (CV of 0.62; PMID: 30333186, 36310225) are classified as having UBQLN2 puncta. The number of cells with diffuse (D) or punctate (P) UBQLN2, as well as the percentage of cells with punctate UBQLN2 (%P) mean CV are summarized for each population. (**C**) The percentage of living neurons showing UBQLN2 puncta over the first 4 days following transfection. Neurons identified as having puncta were those with a Cy5-CV above the calculated threshold of 0.62. Error bars show SEM for 3 replicate experiments. A two-way ANOVA demonstrated a main effect of genotype (F = 47.298, *p* = 2E−16) and time (F = 4.803, *p* = 0.0038) as well as an interaction (F = 2.736, *p* = 0.000948). * indicates *p* < 0.05 calculated by Tukey’s post-hoc test in pairwise comparisons with WT at the same timepoint. (**D**) Relative risk of UBQLN2 puncta formation as a function of UBQLN2 expression level. Within each population, neurons were binned into 5 quintiles of expression based on their day 1 Cy5 fluorescent intensity values. For each population, Cox proportional hazards analysis was performed using the lowest quintile (0–20%) as the reference population. The hazard ratio (relative risk of puncta formation) for each quintile relative to the reference population is plotted on the y-axis. Hazard ratios were derived from three pooled experiments stratified by experiment date. * indicate a higher hazard ratio with a *p* < 0.05 for the highest quintile versus the lowest. (**E**) Relative risk of puncta formation for UBQLN2 variants. In each of three experimental replicates, neurons across all seven groups were binned based on day 1 Cy5 fluorescent intensity. Within each expression quintile the relative risk of UBQLN2 puncta formation was compared across genotypes. Hazard ratios and statistical significance are reported in Table 3. * indicates *p* < 0.05. (**F**) Fluorescence microscopy shows the indicated recombinant UBQLN2 proteins phase-separating into micron-sized droplets in vitro, when incubated for 10 min at 30 °C using 75 μM protein in 20 mM Hepes, 200 mM NaCl and 1 mM DTT (pH 7). Scale bar, 5 μM. (**G**) Results from spectrophotometric turbidity assay as a function of temperature, comparing LLPS of the indicated WT or mutant UBQLN2 proteins using 60 μM protein in 20 mM Hepes, 200 mM NaCl and 1 mM DTT (pH 7). (**H**) SDS-PAGE gel showing the purity of recombinant UBQLN2 proteins.

To determine whether the location of a UBQLN2 mutation affects self-assembly, and whether different amino acid substitutions at the same site result in different self-assembly propensities, we used automated microscopy^18,30–32^ to analyze the aggregation of each UBQLN2 mutant in neurons over several days (Fig. 3). We transfected primary rat cortical neurons with near-infrared fluorescent protein (iRFP) or iRFP-tagged UBQLN2 variants and co-expressed mApple to serve as a morphology marker (Fig. 3A). As before^18^, we calculated the coefficient of variation (CV) for iRFP-tagged UBQLN2 variants as a quantitative indicator of aggregation^33^. Neurons displaying a CV above the aggregate threshold were classified as having UBQLN2 aggregates (Fig. 3B). Based on previous work^18^ as well as the internal mobility of UBQLN2 puncta in HEK293 cells (Fig. 2), UBQLN2 puncta in transfected neurons appear to comprise a mixture of liquid-like condensates, hydrogel-like structures, and aggregated protein. Accordingly, for simplicity we will refer to accumulations of UBQLN2 fluorescence as “puncta” to represent all of these possible structures.

**Table 3:** Relative risk of puncta formation across genotypes.

Group	N	Hazard ratio	Lower 95%	Upper 95%	*p* value
WT	1227	1	1	1	1
282V	1693	0.7909	0.6823	0.9169	0.002
446R	1155	0.7497	0.6323	0.8889	0.001
497H	1855	1.085	0.946	1.2444	0.243
497S	1804	0.6521	0.5576	0.7625	8.57E-08
P506T	1491	1.6306	1.4283	1.8615	4.63E-13

While iRFP alone remained diffuse, iRFP-tagged UBQLN2 variants formed puncta throughout the soma and neuritic processes of transfected neurons. The percentage of neurons developing UBQLN2 puncta over the first 4 days following transfection was calculated and plotted as a function of days in culture (Fig. 3C). Across all 5 UBQLN2 mutants, only P506T showed a significantly higher risk of puncta formation than WT while A282V, M446R and P497S were significantly less likely to form puncta than WT (Fig. 3B, C, Table 3). Since the expression level of UBQLN2 affects its self-assembly properties^18,33^ we plotted the relative risk of puncta formation, calculated using Cox proportional hazard analysis, as a function of single-cell UBQLN2-iRFP fluorescence intensity, which is directly proportional to the expression of UBQLN2^30,34,35^ (Fig. 3D). Within each expression quintile we observed the same trends in puncta formation indicating that differences in puncta propensity are due to intrinsic properties of the UBQLN2 protein itself, not expression level. When restricting our comparison to neurons with the same UBQLN2 expression level across genotypes (Fig. 3E), the cumulative risk of puncta formation over 4 days was greatest for UBQLN2-P506T, as expected, but lower for all other UBQLN2 mutants.

To assess self-assembly properties of each UBQLN2 mutation in vitro, recombinant full-length UBQLN2-WT, UBQLN2-A282V, UBQLN2-P497H, UBQLN2-P497S and UBQLN2-P506T proteins were purified from bacteria (Fig. 3H) and incubated for 10 min at 30 °C, during which time all tested UBQLN2 proteins phase-separated into micron-sized droplets (Fig. 3F). We then varied the temperature and compared the degree of LLPS for different UBQLN2 mutants using a spectrophotometric turbidity assay (Fig. 3G). All 4 mutant UBQLN2 constructs showed a range of turbidity depending on the incubation temperature with the highest level of turbidity occurring in the 36–48 °C range. UBQLN2-P506T showed the highest degree of turbidity, mirroring its more robust puncta formation in cells. UBQLN2-P506T also phase separated at a lower temperature compared to WT and other mutants. Like UBQLN2-P506T, UBQLN2-P497H formed droplets at a lower temperature than WT, but also exhibited lower turbidity at higher temperatures than other genotypes, including WT. In transfected neurons UBQLN2-P497H was second most likely to form aggregates after UBQLN2-P506T (Fig. 3), but also showed the most rapid recovery in the FRAP experiments (Fig. 2). Together, these observations indicate that UBQLN2-P497H may be more dynamic within condensates than other genotypes, including UBQLN2-WT.

### Effect of UBQLN2 mutations on neuronal toxicity

**Figure 4:**
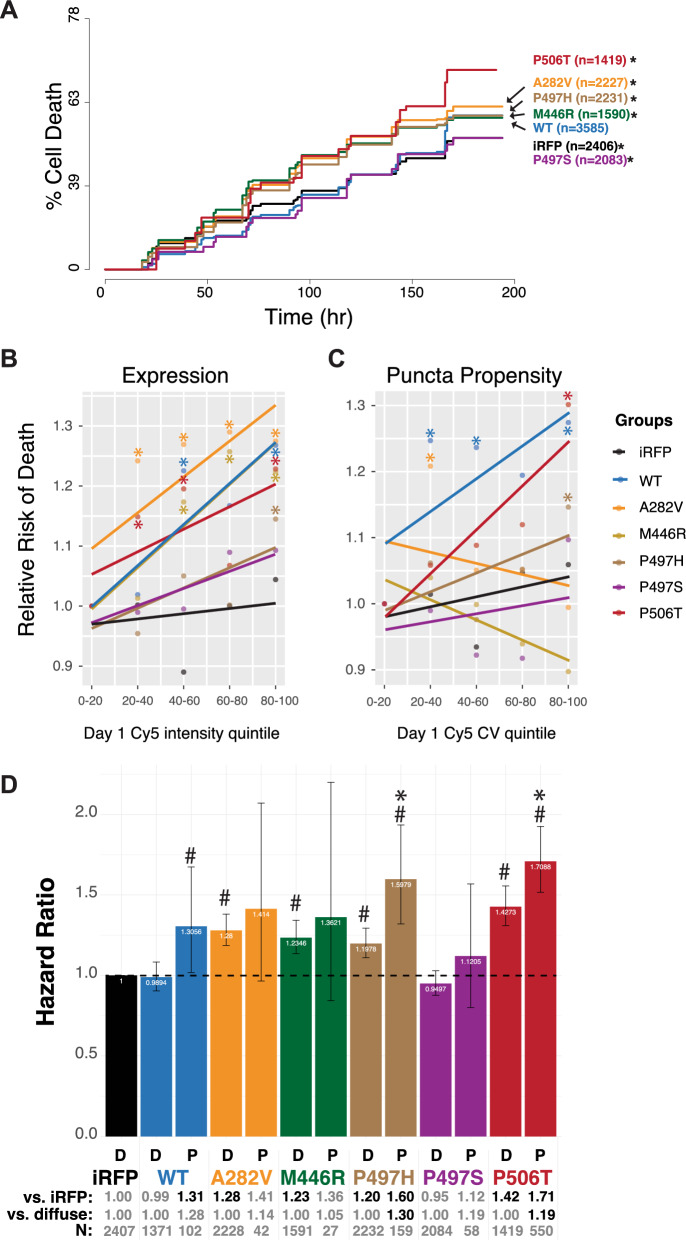
Toxicity is inconsistently associated with UBQLN2 puncta. (**A**) Neuronal survival measured by automated fluorescence microscopy. The survival of rat primary cortical neurons transfected with mApple and each iRFP-tagged UBQLN plasmid was measured using Cox proportional hazards analysis. Table 4 reports hazard ratios, confidence intervals, *p* values and number of neurons per group using iRFP as the reference population. Table 5 instead uses UBQLN2-WT as the reference population. (**B**, **C**) Relative risk of death as a function of UBQLN2 expression and puncta forming propensity. In each population, neurons were stratified into quintiles based on day 1 Cy5 fluorescence intensity (expression) or Cy5 CV (puncta propensity). Within each population the survival of each quintile was compared using Cox proportional hazards analysis with the lowest expressing quintile serving as the reference group. Hazard ratios, confidence intervals, and p-values are reported in Tables 6 (expression) and 7 (punctateness). * denotes *p* < 0.05 for cox proportional hazards analysis using the lowest quintile as a reference population. (**D**) Comparison of survival in neurons with diffuse versus punctate UBQLN2. Within each population, neurons were divided into those cells that had diffuse (D) or punctate (P) UBQLN2 on the first day after transfection. Survival of each population was then measured via longitudinal microscopy and Cox proportional hazards analysis. Reported results are from three pooled experimental replicates. The column plot depicts the hazard ratio of each group compared to iRFP. Error bars represent 95% confidence intervals. Hazard ratios and sample size are reported, with p-values and confidence intervals also reported in Table 8. For each UBQLN2 variant, the hazard ratio of puncta-containing neurons is also reported using neurons with diffuse UBQLN2 as the reference control. Hazard ratios, *p* values, and sample sizes for these analyses are reported in Table 9. Bolded values, *p* < .05 using Cox proportional hazards analysis. # signifies *p* < 0.05 using Cox proportional hazards comparing to iRFP, * is *p* < 0.05 relative to diffuse of the same genotype.

We previously observed that both aggregation propensity and abundance of UBQLN2-WT and UBQLN2-P506T strongly correlated with neuronal toxicity^18^. Here, we extended that analysis to other UBQLN2 mutants. Rat primary cortical neurons were again transfected with iRFP alone, or iRFP-tagged UBQLN2 variants. Automated fluorescence microscopy was used to measure neuronal survival, and the risk of cell death was compared among conditions using Cox proportional hazards analysis (^36^; Fig. 4A and Tables 4, 5). As before, UBQLN2-WT expression increased the risk of death over iRFP alone. Compared to UBQLN2-WT, UBQLN2-P506T was significantly more toxic, increasing risk of death by more than 60%. Surprisingly, no other pathogenic UBQLN2 mutants were more toxic than UBQLN2-WT, and UBQLN2-P497S was even slightly less toxic than UBQLN2-WT.

**Table 4:** Relative risk of neuronal cell death relative to iRFP.

Group	N	Hazard ratio	Lower 95%	Upper 95%	*p* value
iRFP	2406	1	1	1	1
WT	3585	1.0775	1.0043	1.156	0.0376105
282V	2227	1.2866	1.1927	1.3879	7.18E−11
446R	1590	1.2392	1.1393	1.3478	5.67E−07
497H	2231	1.2045	1.1156	1.3005	1.98E−06
497S	2083	0.9539	0.8806	1.0334	0.2475927
P506T	1419	1.4332	1.3157	1.5612	1.64E−16

**Table 5:** Relative risk of neuronal cell death relative to UBQLN2-WT.

Group	N	Hazard ratio	Lower 95%	Upper 95%	*p* value
WT	3585	1	1	1	1
282V	2227	1.1627	1.0869	1.2438	1.18E−05
446R	1590	1.1088	1.0272	1.197	0.00811025
497H	2231	1.0917	1.0199	1.1686	0.01150332
497S	2083	0.8555	0.7961	0.9193	2.12E−05
P506T	1419	1.3459	1.2491	1.4503	6.30E−15

**Table 6:** Relative risk of cell death in UBQLN2 expressing neurons broken down by expression quintile.

Groups	ntile	Hazard	*p*	Lower95	Upper95
iRFP	1	1	1	1	1
iRFP	2	1.0018	0.979179	0.8732	1.1494
iRFP	3	0.8901	0.102119	0.7741	1.0234
iRFP	4	1.0004	0.995295	0.8719	1.1479
iRFP	5	1.0442	0.535395	0.9107	1.1972
WT	1	1	1	1	1
WT	2	1.019	0.844543	0.8445	1.2294
**WT**	**3**	**1.2254**	**0.028668**	**1.0214**	**1.4702**
WT	4	1.1673	0.098805	0.9714	1.4028
**WT**	**5**	**1.268**	**0.01011**	**1.0581**	**1.5195**
282V	1	1	1	1	1
**282V**	**2**	**1.242**	**0.002216**	**1.081**	**1.427**
**282V**	**3**	**1.2693**	**0.000732**	**1.1053**	**1.4577**
**282V**	**4**	**1.29**	**0.000318**	**1.123**	**1.4818**
**282V**	**5**	**1.2748**	**0.0006**	**1.1097**	**1.4645**
446R	1	1	1	1	1
446R	2	1.0131	0.881116	0.8545	1.2011
**446R**	**3**	**1.1735**	**0.059321**	**0.9937**	**1.3857**
**446R**	**4**	**1.2573**	**0.006598**	**1.0658**	**1.4831**
**446R**	**5**	**1.2238**	**0.016899**	**1.0369**	**1.4444**
497H	1	1	1	1	1
497H	2	0.9543	0.507765	0.8309	1.096
497H	3	1.0502	0.480976	0.9164	1.2035
497H	4	1.0016	0.981518	0.8733	1.1488
**497H**	**5**	**1.145**	**0.048026**	**1.0012**	**1.3095**
497S	1	1	1	1	1
497S	2	0.9896	0.89391	0.8492	1.1533
497S	3	0.9952	0.950567	0.8535	1.1603
497S	4	1.0896	0.264385	0.9371	1.267
497S	5	1.0928	0.251147	0.9391	1.2715
P506T	1	1	1	1	1
**P506T**	**2**	**1.1488**	**0.045103**	**1.003**	**1.3158**
**P506T**	**3**	**1.1953**	**0.009949**	**1.0437**	**1.3689**
P506T	4	1.0677	0.347184	0.9314	1.2241
**P506T**	**5**	**1.2284**	**0.002723**	**1.0738**	**1.4053**

Significant values are in bold.

**Table 7:** Relative risk of cell death in UBQLN2 expressing neurons broken down by CV_iRFP_ quintile.

Groups	ntile	Hazard	*p*	Lower95	Upper95
iRFP	1	1	1	1	1
iRFP	2	1.0143	0.839962	0.8837	1.1642
iRFP	3	0.9346	0.339932	0.8135	1.0738
iRFP	4	1.0469	0.513145	0.9126	1.201
iRFP	5	1.0592	0.41054	0.9236	1.2147
WT	1	1	1	1	1
**WT**	**2**	**1.2469**	**0.019083**	**1.0368**	**1.4996**
**WT**	**3**	**1.2365**	**0.025273**	**1.0267**	**1.4892**
WT	4	1.1944	0.061107	0.9918	1.4385
**WT**	**5**	**1.2743**	**0.009889**	**1.06**	**1.5321**
282V	1	1	1	1	1
**282V**	**2**	**1.2083**	**0.005647**	**1.0568**	**1.3816**
282V	3	1.0507	0.477304	0.9168	1.2041
282V	4	1.0512	0.472977	0.9172	1.2047
282V	5	0.9945	0.937966	0.8664	1.1417
446R	1	1	1	1	1
446R	2	1.0395	0.639679	0.8838	1.2226
446R	3	0.9989	0.98905	0.8486	1.1758
446R	4	0.9393	0.45602	0.7967	1.1074
446R	5	0.8974	0.202373	0.7598	1.0599
497H	1	1	1	1	1
497H	2	1.0616	0.386766	0.9272	1.2154
497H	3	0.9761	0.73058	0.8506	1.1201
497H	4	1.0522	0.463258	0.9184	1.2056
**497H**	**5**	**1.1464**	**0.045877**	**1.0025**	**1.3109**
497S	1	1	1	1	1
497S	2	0.9897	0.892195	0.8519	1.1498
497S	3	0.9223	0.295602	0.7926	1.0733
497S	4	0.9175	0.269411	0.7874	1.069
497S	5	1.0969	0.219793	0.9462	1.2716
P506T	1	1	1	1	1
P506T	2	1.0583	0.41508	0.9235	1.2128
P506T	3	1.0884	0.221435	0.9502	1.2468
P506T	4	1.1197	0.103311	0.9773	1.2828
**P506T**	**5**	**1.3014**	**0.000116**	**1.1382**	**1.4879**

Significant values are in bold.

**Table 8:** Relative risk of cell death in neurons with diffuse or punctate UBQLN2 (iRFP as reference).

Group	N	Hazard ratio	Lower 95%	Upper 95%	*p* value
1 iRFP	2407	1	1	1	1
2 WT	1371	0.9894	0.9038	1.0832	0.81834168
**2 WT_aggT1**	**102**	**1.3056**	**1.0184**	**1.6739**	**0.03541169**
**3 282V**	**2228**	**1.28**	**1.1866**	**1.3807**	**1.69E−10**
3 282V_aggT1	42	1.414	0.9656	2.0706	0.07503873
**4 446R**	**1591**	**1.2346**	**1.1351**	**1.3428**	**8.77E−07**
4 446R_aggT1	27	1.3621	0.8436	2.1994	0.20613535
**5 497H**	**2232**	**1.1978**	**1.1095**	**1.2932**	**3.90E−06**
**5 497H_aggT1**	**159**	**1.5979**	**1.3199**	**1.9346**	**1.54E−06**
6 497S	2084	0.9497	0.8767	1.0288	0.20605645
6 497S_aggT1	58	1.1205	0.8007	1.5682	0.50697825
**7 P506T**	**1419**	**1.4273**	**1.3092**	**1.5561**	**6.95E−16**
**7 P506T_aggT1**	**550**	**1.7088**	**1.5166**	**1.9254**	**1.38E−18**

Significant values are in bold.

**Table 9:** Relative risk of cell death in neurons with diffuse or punctate UBQLN2 (diffuse as reference).

Group	N	Hazard ratio	Lower 95%	Upper 95%	*p* value
WT	1371	1	1	1	1
WT_aggT1	102	1.282	0.9912	1.6581	0.0584191
A282V	2228	1	1	1	1
A282V_aggT1	42	1.1394	0.7773	1.67	0.50364426
M446R	1591	1	1	1	1
M446R_aggT1	27	1.0496	0.6451	1.7077	0.84537185
P497H	2232	1	1	1	1
**P497H_aggT1**	**159**	**1.2978**	**1.0642**	**1.5828**	**0.01005345**
P497S	2084	1	1	1	1
P497S_aggT1	58	1.1916	0.8477	1.675	0.3131055
P506T	1419	1	1	1	1
**P506T_aggT1**	**550**	**1.1927**	**1.0582**	**1.3443**	**0.00388385**

Significant values are in bold.

We then tested whether expression level (Fig. 4B) or puncta propensity (Fig. 4C) correlated with neuronal toxicity in neurons expressing iRFP-tagged UBQLN2 variants. In each population, neurons were stratified into quintiles based on UBQLN2 expression on day 1, judged by iRFP (Cy5) fluorescence intensity, or CV (puncta propensity). Within each population, the survival of each quintile was compared using Cox proportional hazards analysis, with the lowest expressing quintile serving as the reference group. Expression level was positively correlated with risk of death for UBQLN2-WT, P506T, M446R and A282V, but not for P497S or P497H (Fig. 4B, Table 6). Only UBQLN2-WT showed a positive correlation between puncta propensity and toxicity, an effect that was observed in most expression quintiles (Fig. 4C, Tables 6, 7).

To further evaluate the toxicity associated with puncta formation, we separately measured cell death in neurons showing diffuse versus punctate UBQLN2 expression patterns (Fig. 4D). Relative to those neurons expressing iRFP alone, neurons with punctate UBQLN2-WT but not diffuse UBQLN2-WT had an elevated risk of death. Diffuse UBQLN2-A282V and UBQLN2-M446R were toxic, with puncta formation not significantly elevating toxicity. Puncta formation increased risk of cell death in neurons expressing UBQLN2-P497H and UBQLN2-P506T. These discrepant effects highlight the inconsistent relationship between self-assembly properties and toxicity across UBQLN2 mutants.

### Pathogenic UBQLN2 mutations do not affect macroautophagy

**Figure 5:**
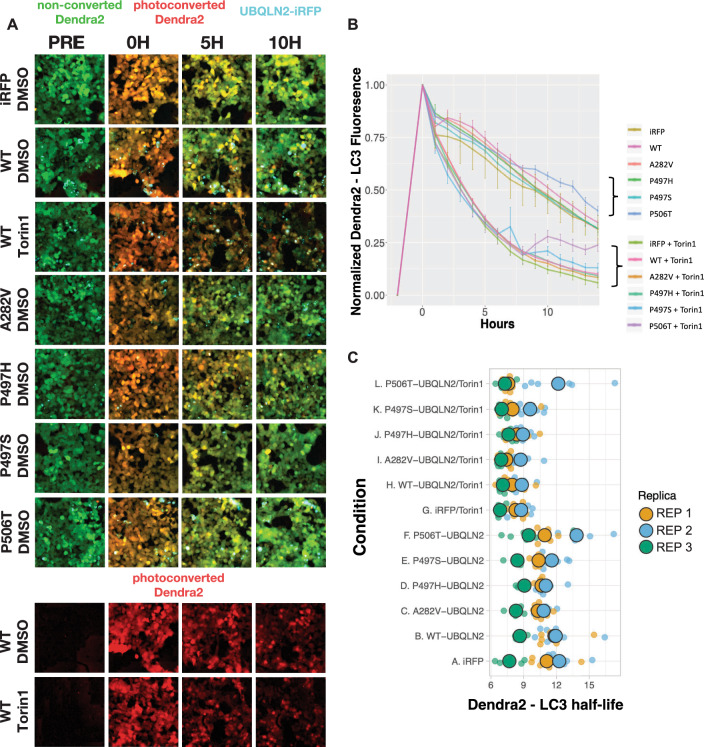
UBQLN2 expression does not impair macroautophagy. (**A**) Representative images of Dendra2-LC3 HEK293 cells transfected with iRFP or UBQLN2-IRFP plasmids and treated with either DMSO or 1 µM Torin1. Cells were imaged in the red (photoconverted Dendra2-LC3), green (unconverted Dendra2-LC3), and Cy5 (cyan- IRFP) channels prior to photoconversion and then at the indicated time points after photoconversion. For cells expressing both WT-UBQLN2-iRFP composite images and the red channel are displayed to highlight decay in photoconverted Dendra2-LC3. (**B**) Representative experiment of Dendra2-LC3 HEK293 cells transfected with the indicated UBQLN2-iRFP plasmids and treated with 1 µM Torin1 or DMSO. Error bars signify standard error of the mean from 6 wells. (**C**) Superplot showing Dendra-LC3 half-life values for the indicated UBQLN2 genotypes with and without Torin1, across three experimental replicates, with 6 wells evaluated/replicate. Each small dot shows a half-life value for one well in one experiment, while large dots depict the mean value for that experiment. All data are summarized in Table 10.

Because UBQLN2 participates in macroautophagy^27–29^, we asked whether pathogenic UBQLN2 mutations disrupt this pathway. We previously created a macroautophagy reporter cell line by CRISPR/Cas9-mediated insertion of the photoconvertible Dendra2 protein into the endogenous *MAP1LC3B* locus of HEK293 cells^37^. These cells enable quantitative estimates of autophagy flux by measuring the time-dependent decay of photoconverted (red) Dendra2-LC3. To determine if mutant UBQLN2 variants impair autophagic flux, we transfected each iRFP-UBQLN2 construct into these reporter cells and measured the half-life of Dendra2-LC3 (Fig. 5A). No significant differences in LC3 turnover were detected with any UBQLN2 construct compared to iRFP expression alone (Fig. 5B, C). To account for the possibility that changes in macroautophagy might not be observable without stimulation, we repeated the assay in the presence of Torin1, a selective ATP-competitive inhibitor of mTOR (mammalian target of Rapamycin) that effectively upregulates autophagy^37,38^. Although Torin1 treatment increased Dendra2-LC3 turnover in comparison to vehicle (Fig. 5), confirming the effect of Torin1 on autophagy flux, we failed to detect any differences in autophagy flux in cells expressing UBQLN2 variants relative to the control (iRFP). These results argue against a strong influence of UBQLN2 over-expression on macroautophagy flux.

**Table 10:** Summary of Dendra2-LC3 half-life experiments.

	Condition	n	Mean	SD	sem
1	A. iRFP	3	10.35	2.38	1.68
2	B. UBQLN2-WT	3	10.8	1.87	1.32
3	C. UBQLN2-A282V	3	9.8	1.34	0.94
4	D. UBQLN2-P497H	3	10.24	1.04	0.73
5	E. UBQLN2-P497S	3	10.12	1.59	1.12
6	F. UBQLN2-P506T	3	11.4	2.23	1.58
7	G. iRFP/Torin1	3	7.95	1	0.71
8	H. UBQLN2-WT/Torin1	3	7.95	0.86	0.61
9	I. UBQLN2-A282V/Torin1	3	7.69	0.92	0.65
10	J. UBQLN2-P497H/Torin1	3	8.31	0.66	0.47
11	K. UBQLN2-P497S/Torin1	3	8.18	1.31	0.93
12	L. UBQLN2-P506T/Torin1	3	9.01	2.73	1.93

## Discussion

A recurring and important question in neurodegenerative diseases is the relevance of aberrant protein accumulation to disease pathogenesis. Whether protein accumulation and aggregation are causal to, or simply a byproduct of, the disease process remains an unanswered question. Arguably the best evidence exists for tauopathies in which tau accumulation and aggregation are tightly linked with neurodegeneration^39–41^. In the case of UBQLN2-mediated neurodegenerative disease, however, the answer is far less clear. Our studies call into question whether aggregation per se is a primary contributor to disease pathogenesis. While we confirmed a correlation between protein accumulation, aggregation and toxicity for one *UBQLN2* mutation, P506T, our evaluation of other mutations did not extend this finding. All the studies conducted here were carried out in cells that still retained endogenous UBQLN2, thereby limiting any observed effects of *UBQLN2* mutations solely to gain-of-function and dominant-negative effects without allowing us to detect loss-of-function effects. Despite this, our observations align with what is known about full-length UBQLN2 phase separation in vitro: in comparison to P506T, other *UBQLN2* mutations appear to have reduced effects on phase separation^42^. In previous in vitro work, we observed that introducing P497 or P506 point mutations into a truncated protein backbone (amino acids 450–624) does affect significantly affects LLPS behavior^42^, but we failed to observe similar consequences using the full-length proteins studied here. Nevertheless, differences in the LLPS behavior of full-length UBQLN2 variants suggest that factors beyond the intrinsic structure of UBQLN2, including altered interactions with UBQLN2 binding partners, may be responsible for these effects. The FRAP data on UBQLN2 variants in cells (Figs. 1, 2) aligns with our earlier research on the dynamics of stress-induced UBQLN2 puncta^43^, suggesting conserved and significant effects on UBQLN2 condensate behavior in vitro and in cellulo.

UBQLN2 possesses an inherent propensity to undergo biomolecular condensation^15–18^. Differences in the extent of condensation may emerge from intrinsic structural differences or changes to UBQLN2’s interactions with other molecules. The differences we observed in the in vitro turbidity assay can only reflect the effects of intrinsic structural differences between WT and mutant UBQLN2, whereas the behavior of UBQLN2 variants in cells, while largely consistent with our in vitro results, could be affected by other proteins, nucleic acids and macromolecules.

In previous work we and others have shown that UBQLN2 phase separation is affected by ubiquitin binding which occurs in the UBA domain. Although none of the UBQLN2 mutations in the current study occur in the UBA domain, it is reasonable to speculate that the mutations, particularly in the PXX domain, could result in allosteric changes to protein structure that alter the ubiquitin binding site. To our knowledge an evaluation of ubiquitin binding affinity to ALS/FTD UBQLN2 mutants has not been done. A future study focused on this question combined with an evaluation of the effect of the ubiquitin-binding deficient mutant L619A on UBQLN2 LLPS would be very informative.

UBQLN2 biomolecular condensates likely play important roles in cellular processes such as stress granule formation or protein turnover via the UPS^15,44^. However, the unique physicochemical properties of condensates, such as their high viscosity and density, can also affect protein folding, stability, and solubility, making UBQLN2 more prone to aggregation and cellular toxicity, as is the case for the P506T mutation. Even so, in our hands UBQLN2 aggregation propensity failed to consistently correlate with toxicity of UBQLN2 variants in neurons. In certain instances, the formation of aggregates can serve as a protective mechanism or a response to cellular stress, sequestering misfolded or damaged proteins and thereby preventing their detrimental effects on cellular function. One potential example is the A282V mutation which forms aggregates less frequently than WT UBQLN2 yet exhibits higher toxicity; the absence of UBQLN2-A282V aggregation may facilitate cell death if the soluble protein is primarily responsible for downstream toxicity. Alternatively, the A282V mutation might decrease UBQLN2’s ability to phase separate and so prevent its critical participation in PQC pathways. This model is consistent with prior data suggesting that disease-associated *UBQLN2* mutations disrupt macroautophagy^27,28^. Unlike WT UBQLN2, mutant UBQLN2 was unable to acidify autophagosomes and promote autophagic flux^27^, suggesting loss-of-function toxicity. The P506T mutation, which has a high tendency to aggregate, may exert its toxicity through a different mechanism. For example, the increased propensity of UBQLN2-P506T to form aggregates might lead to dominant-negative effects, with sequestration of the entire functional pool of UBQLN2 molecules in the cell and subsequent loss of function. Our study, however, argues against dominant-negative properties of mutant UBQLN2 as a generalized pathogenic mechanism.

While we did not observe any changes in macroautophagy in cells expressing mutant UBQLN2, these cells still express endogenous UBQLN2, which presumably functions in autophagy. We attribute this discrepancy to differences in model systems used across studies. Indeed, we confirmed changes in levels of autophagy-related proteins in cells over-expressing UBQLN2-P497S and UBQLN2-P506T compared to UBQLN2-WT^29^. To reconcile the divergent findings on autophagy, aggregation and toxicity of mutant UBQLN2, investigations of additional physiologically relevant model systems and reporter assays will be needed.

A limitation of our study is the fact that we introduced UBQLN2 mutants into primary neurons and cell lines that still contain the endogenous, wildtype UBQLN2. This approach restricts our ability to thoroughly assess the negative effects and functionality of UBQLN2 mutations. Moreover, UBQLN2 overexpression itself changes LLPS behavior and aggregation propensity even in the absence of mutation, complicating the interpretation of the data^18^. While we took expression levels into account in the analysis of puncta propensity and toxicity, even small differences in UBQLN2 expression levels across genotypes can make direct comparisons difficult. To address this limitation, it will be important to conduct further investigations using alternative models, including knockout cell lines and knock-in transgenic animals. Future studies using such models may uncover potential loss-of-function phenotypes associated with *UBQLN2* mutations under conditions that closely resemble endogenous UBQLN2 expression levels. We expect that these investigations will be crucial for gaining a more comprehensive understanding of phenotypes associated with UBQLN2 variants and their implications for neurodegeneration.

Despite these limitations, our data support a model in which *UBQLN2* mutations are not functionally equivalent. As such, future efforts to therapeutically target UBQLN2 in ALS/FTD may not be universally applicable to all mutations. Instead, a more directed and individualized strategy may be required for each *UBQLN2* mutation.

## Materials and methods

### In vivo fluorescence recovery after photobleaching

HEK293 cells were plated at 80% cell density onto 4-well LAB-TEK borosilicate chambers (ThermoFisher). HEK293 cells were obtained directly from ATCC (ATCC CRL-1573) and used at passage numbers < 25. HEK293 cells were cultured in DMEM media supplemented with 10% FBS and antibiotic/antimicotic. On the day after plating cells were co-transfected with 500ng of pGW1-mApple and 500ng pGW1-GFP-UBQLN2 constructs (UBQLN2-WT, UBQLN2-A282V, UBQLN2-M446R, UBQLN2-P497S, UBQLN2-P497H, UBQLN2-P506T, UBQLN2-L619A). Cells were imaged between 24 and 48 h post transfection using a Nikon A1 confocal microscope equipped with a 60 × objective controlled by Nikon Elements software. Prior to bleaching, cells were imaged at 30 Hz for 10 s. A circular region of interest (ROI) was generated in Elements with a radius of 3.7 µm and photobleached with 7 1 s pulses with a 488 nm laser at 30% power. Immediately following bleaching, cells were imaged every 5 s for 1 min, and then subsequently every 10 s for 4 min.

Image processing and analysis was performed using FIJI. To prevent the drift of granules over time in image stacks, “rigid body” stack registration was performed. Using the images acquired prior to photobleaching, a whole-granule ROI was generated. At each timepoint the percent recovery was quantified as ROI_bleached_ GFP integrated density/ROI_whole-granule_ GFP integrated density, with the average pre-bleach values set to 1, and the value from the first post-bleach image set to 0.

Each recovery curve was fit to the equation y(t) = A(1 − e^−τt^), where t = time, A = peak recovery and τ = time constant^82^. Time to half maximal recovery (T_1/2_ max) was measured using the equation T_1__/2_ = ln(0.5)/− τ. We calculated the diffusion coefficient (D) for each granule using the equation D = (0.88 ROI_bleached_ radius^2^)/4 T_1/2_ and viscosity (η) using the Einstein-Stokes equation D = k_B_T/6πηr where k_B_ = Boltzmann constant, T = temperature in K, r = Stokes radius of the particle. We calculated the Stokes radius of 4.5nm for GFP-UBQLN2 using the equation R_min_ = (0.66 molecular weight)^1/3^, where Rmin is the minimum radius of a sphere bounding a globular protein with a molecular weight of M^10^.

### Primary neuron culture and transfection:

Culture and transfection of rat primary cortical neurons was performed as previously described. Briefly, embryonic day 19–20 rat embryos were dissected and dissociated and plated at a density of 6 × 10^5^ cells/mL in 96 well plates. On in vitro day 4 (DIV4) neurons were transfected with 100ng of pGW1-mApple and 100ng of different pGW1-iRFP-UBQLN2 constructs (UBQLN2-WT, UBQLN2-A282V, UBQLN2-M446R, UBQLN2-P497S, UBQLN2-P497H, UBQLN2-P506T), using Lipofectamine 2000. Following transfection neurons were cultured in Neumo photostable media (M07-500, Cell Guidance systems, Cambridge UK).

### Automated fluorescence microscopy

Primary neurons were longitudinally imaged as previously described^18^ using a Nikon Eclipse TiE-2000 microscope equipped with a PerfectFocus3 system, a 20 × objective lens, a Brightline Semrock filter set, a Lambda 421 LED illumination system (Sutter Instrument) and an Andor Zyla 4.2 (+) sCMOS camera (Oxford Instruments). Filter turret control and automated plate movements were controlled via µManager software. Assignment of single-cell barcodes and cell-death scoring was achieved as previously described using custom-built software^45^. The suitability of using Cy5 CV_intensity_ was previously established^18,33^. Cy5 CV_intensity_ threshold values used for puncta classification and the percentage of neurons containing puncta over time were quantified using a Python script available at https://github.com/BarmadaLab/puncta_id.

### Differential solubility assay

Differential solubility assays were performed similar to our previous work^30^. HEK cells transfected with each GFP-UBQLN2 construct were rinsed in ice-cold PBS 24 h after transfection. Ice-cold RIPA buffer with protease inhibitors were added to each plate and incubated on ice for 10 min. Each plate was gently scraped with a cell scraper and samples were transferred to pre-chilled conical tubes. Cells were sonicated at 50% amplitude for 5 s 2x, chilled on ice between each sonication step. Samples were centrifuged at 28,000*g* for 15 min at 4 °C to pellet RIPA-insoluble material, with the supernatant removed and saved as the RIPA-soluble fraction. The RIPA-insoluble pellet was washed in RIPA once, and contents resuspended vigorously in urea buffer (7 M urea, 2 M thiourea, 4% CHAPS, 30 mM Tris, pH 8.5). Samples were again centrifuged at 28,000*g* for 15 min at 4 °C, and the supernatant was saved as the RIPA-insoluble, urea-soluble fraction.

### Immunoblotting

6 × Laemmli buffer containing 100 mM DTT was diluted to 1 × in each RIPA-soluble and urea-soluble fraction. Equal volumes of each RIPA sample or urea sample were loaded on acrylamide NuPAGE Novex 4–12% Bis–Tris Protein Gels (Invitrogen) with NuPAGE MES SDS Running Buffer (Invitrogen) and were resolved by gel electrophoresis. Gels were subsequently transferred onto 0.2 μM nitrocellulose membranes at 100 V for 1 h. Membranes were immediately rinsed with deionized H2O, stained with Ponceau S and imaged on a G Box Mini imager (Syngene). Membranes were rinsed with 1 × Tris-Buffered Saline, 0.1% (TBST) for 10 min, and blocked with 5% non-fat dry milk (DotScientific) and 0.05% BSA (Fisher) in 1 × TBST for 1 h. Membranes were incubated by rocking overnight at 4 degrees with α-UBQLN2 (Novus Biologicals NBP2-25,164, 1:1000) or α-GAPDH (EMD Millipore, MAB374, 1:5000) primary antibodies diluted with 5% milk and 0.05% BSA in 1 × TBST). Membranes were rinsed three times with 1 × TBST for 10 min, incubated at room temperature with goat-α-mouse HRP-conjugated secondary antibody (Jackson ImmunoResearch #115-035-146, 1:2000 for α-UBQLN2 blot and 1:5000 for α-GAPDH blot) for 1 h, and then rinsed three times with 1 × TBST for 10 min prior to developing with Western Lightning® Plus ECL (Fisher) or EcoBright Nano/Femto HRP 50 (Innovative Solutions) using the G Box Mini imager set to ECL auto exposure. GeneSys (Syngene) was used for blot quantification.

### Subcloning, protein expression, and purification

UBQLN2 mutants used in spectrophotometric absorbance/turbidity measurements were generated from full length UBQLN2 using Phusion Site-Directed Mutagenesis Kit (Thermo Scientific). UBQLN2 and all the mutants were expressed and purified as described^15^. Briefly, the constructs were expressed in E. coli Rosetta 2 (DE3) pLysS cells in Luria–Bertani (LB) broth at 37 °C overnight. Bacteria were pelleted, frozen, lysed, then purified via a “salting out” process. NaCl was added to the cleared lysate to the final concentration of 0.5 M. UBQLN2 droplets were pelleted and then dissolved in 20 mM Hepes, 1 mM DTT (pH 7). Leftover NaCl was removed through HiTrap desalting column (GE Healthcare). SDS-PAGE gels were performed to confirm the purity of the proteins. Purified proteins were frozen at − 80 °C. No expression for UBQLN2 M446R was observed for several expression temperatures, *E. coli* expression strains, growth media and plasmids containing UBQLN2 M446R gene.

### Spectrophotometric absorbance/turbidity measurements

Protein samples were prepared by adding protein (from stock to a final concentration of 60 μM) to cold Hepes buffer (pH 7, 20 mM Hepes, 1 mM DTT) containing 200 mM NaCl and were kept on ice for at least 5 min before the assay. Absorbance at 600 nm was recorded as a function of temperature on an Agilient Cary 3500 UV/Vis spectrophotometer using a temperature ramp rate of 1 °C/min increasing from 16 to 60 °C. Net absorbance values were recorded after subtracting the absorbance value of a buffer control. Results were averaged from data collected using proteins from at least two separate preps and three trials for each (total n ≥ 6). Data were plotted using Mathematica (Wolfram Research).

### Fluorescence imaging of phase separation

UBQLN2 constructs were prepared to contain 75 μM protein (spiked with DyLight 650-labeled UBQLN2 at 1:1000 molar ratio) in 20 mM Hepes, 200 mM NaCl, and 1 mM DTT (pH 7). Samples were added to MatTek glass bottom dishes that had been coated with 5% bovine serum albumin (BSA) to minimize changes as a result of surface interactions, and incubated at 30 °C. Phase separation was imaged on an ONI Nanoimager (Oxford Nanoimaging Ltd, Oxford, UK) equipped with a Hamamatsu sCMOS ORCA flash 4.0 V3 camera using an Olympus 100 Å ~ /1.4 N.A. objective. Images were prepared using Fiji^1^ and FigureJ plugin^2^.

### Macroautophagic flux measurement via Dendra2-LC3 optical pulse labelling

Dendra2-LC3 HEK293 cells (PMID: 34303705) were transfected with 100ng of iRFP-UBQLN2 plasmids using lipofectamine 2000 (Invitrogen). 24 h after transfection, cells were imaged in the TRITC channel to measure background fluorescence (T_0_) prior to photoconversion with a 4 s DAPI pulse. Immediately after, cells were imaged again in the TRITC channel to measure post-conversion fluorescence intensity (T_1_). Cells were then treated with either 1 µM Torin1 or DMSO and subsequently imaged every hour for 14 h. Fluorescence intensity was normalized to the post-photoconversion intensity of Dendra2-LC3, using the equation: Normalized TRITC intensity_Tx_ = TRITC_Tx_/(TRITC_T1 _− TRITC_T0_). Half-life was calculated using the equation y = e^−kt^. Superplot depicting the variability in both technical and biological replicates was produced using https://huygens.science.uva.nl/SuperPlotsOfData/.

### Ethics approval

All animal experiments were conducted under the approval of The University of Michigan Institutional Animal Care and Use Committee, protocol number PRO00010103. All work was conducted in accordance with the Guide for the Care and Use of Laboratory Animals as adopted by the NIH. All methods are reported in accordance with ARRIVE guidelines.

### Consent to participate

Not applicable.

### Consent for publication

Not applicable.

### Supplementary Information

Supplementary Information.

## Data Availability

The authors confirm that the data supporting the findings in this are available within the article, at repository links provided within the article, and within its supplementary files. The authors agree to share reagents, cell lines and animal models used in this study upon request. Please contact corresponding author Lisa M Sharkey for data or resource requests.
