# Supplementary material for: Pathogenic mutations in UBQLN2 exhibit diverse aggregation propensity and neurotoxicity
Source: Sci Rep. 2024 Mar 13;14:6049. doi: 10.1038/s41598-024-55582-9 (PMC10933299; doi:10.1038/s41598-024-55582-9)

Supplementary Figures

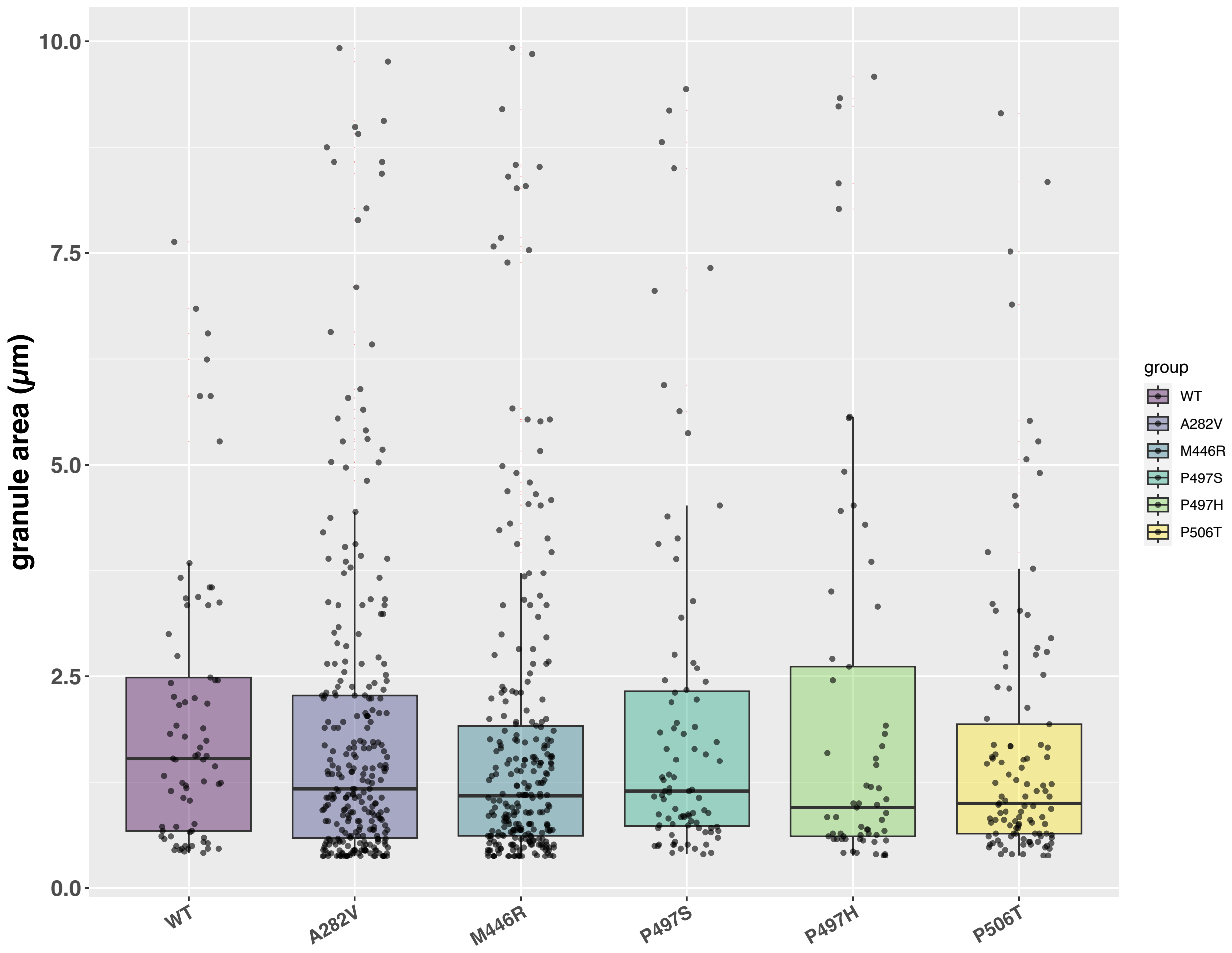

Figure S1. UBQLN2 mutations have no consistent effect on granule size.
Granules analyzed in Figure 2A-C were analyzed for size and compared across UBQLN2 genotypes. The boxplot summarizes granule size across groups. A one-way ANOVA found that the relationship between granule size and genotype was not significant F=.792, p=.55.

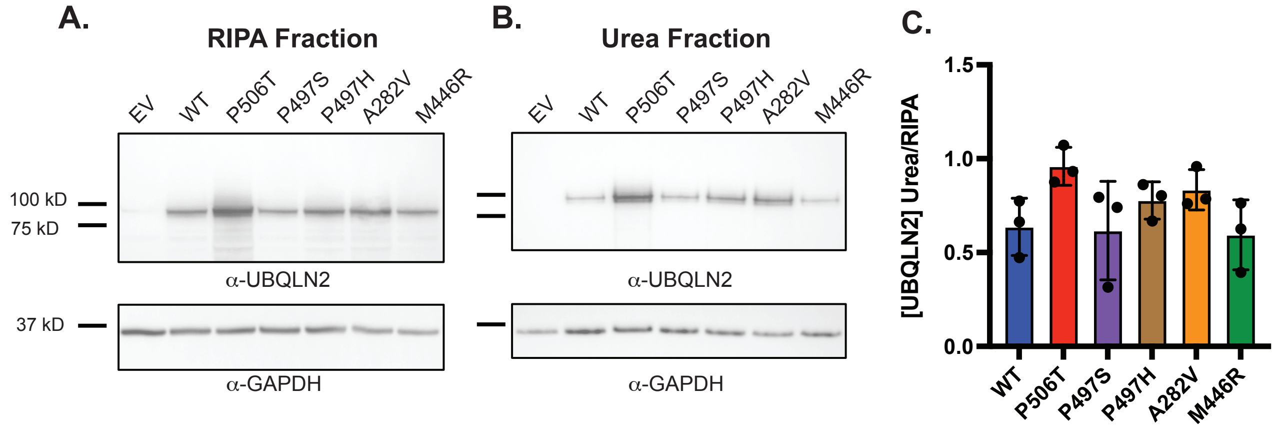

Figure S2. UBQLN2 mutations do not affect detergent solubility.
(A and B) Representative immunoblot to assess the solubility of WT and mutant UBQLN2 in RIPA **(A)** or urea **(B)** buffers. UBQLN2 bands were quantified and the ratio of urea-soluble:RIPA-soluble fractions were quantified from 3 separate experiments and in **(C)**. No significance differences were detected among genotypes by one-way ANOVA.

Uncropped Western Blot images for data in Figure S2.

<u>RIPA Fraction, α-UBQLN2</u>

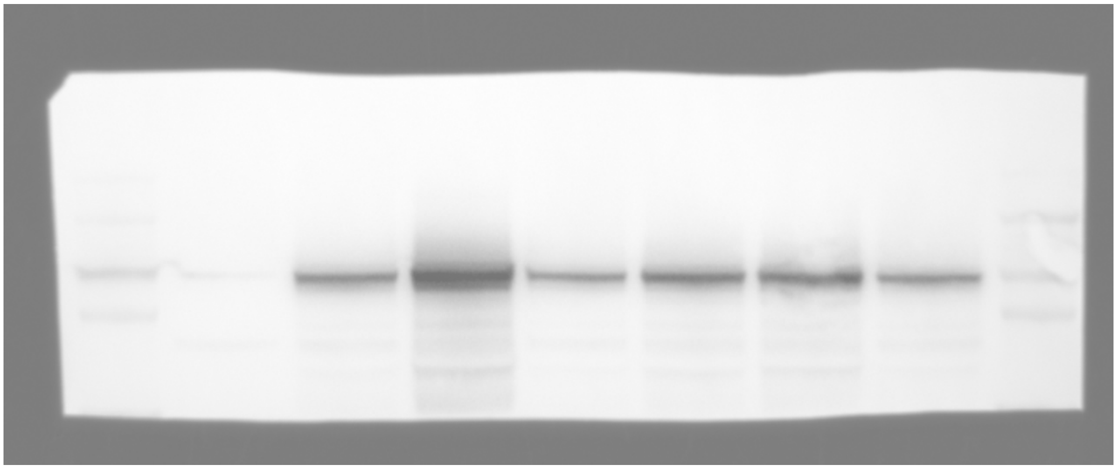

RIPA Fraction, α-GAPDH:

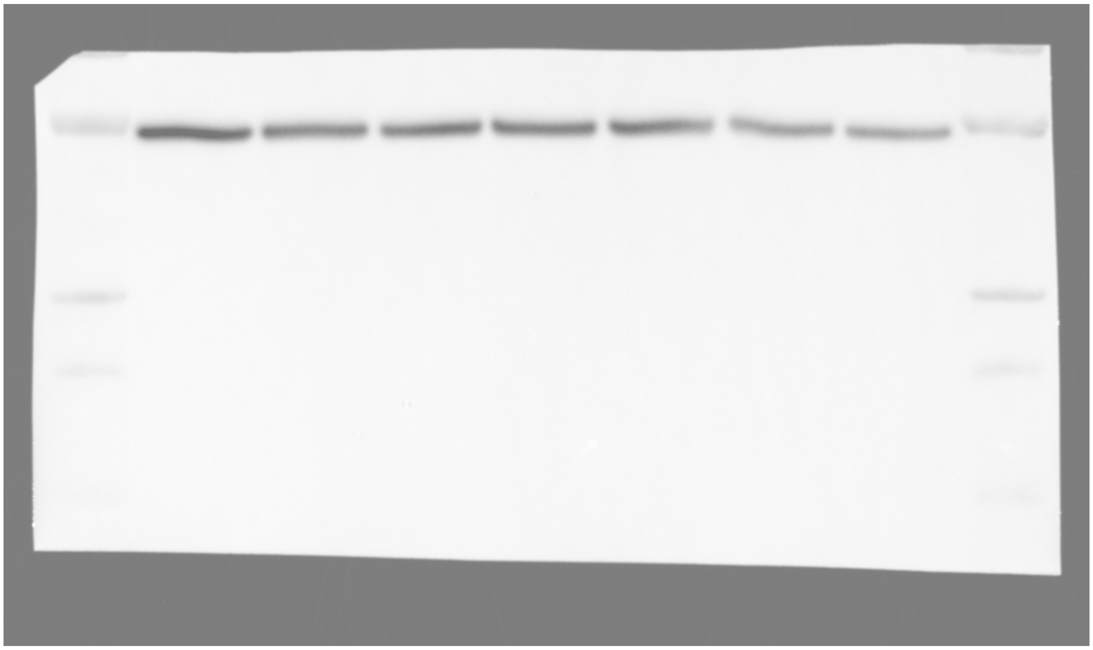

Urea Fraction, α-UBQLN2:

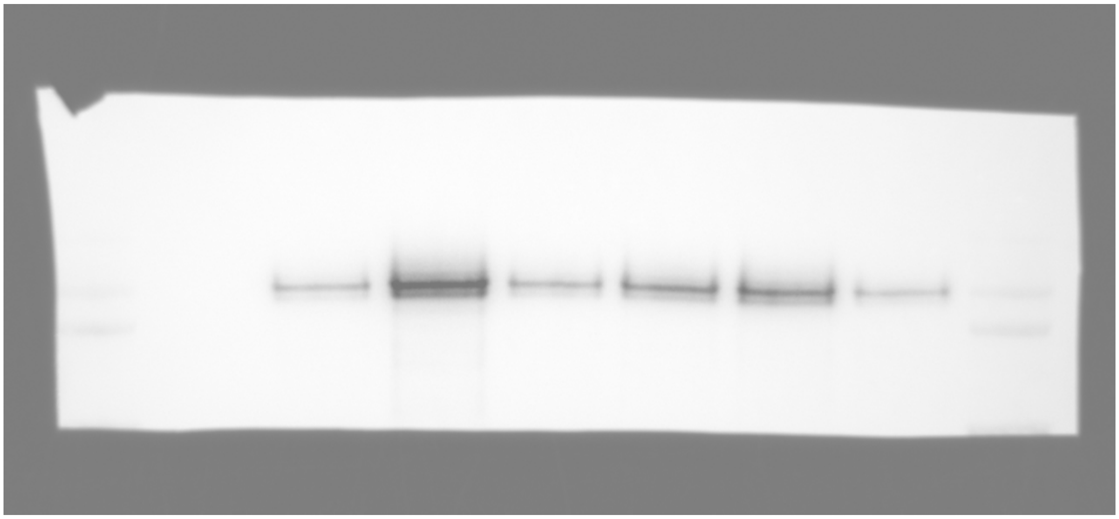

Urea Fraction, α-GAPDH:

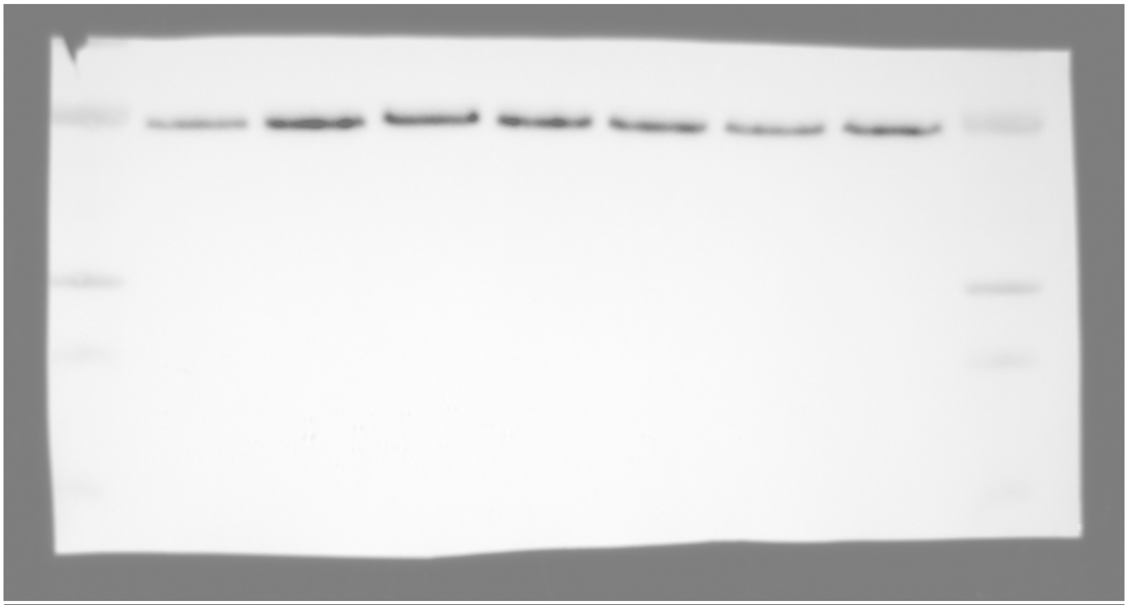

Supplement: Supplementary file 1 — Supplementary Information. [file 41598_2024_55582_MOESM1_ESM.pdf]
